# Use of inert gas jets to measure the forces required for mechanical gene transfection

**DOI:** 10.1186/1475-925X-11-67

**Published:** 2012-09-10

**Authors:** Guillaume Chouinard-Pelletier, Mathieu Leduc, David Guay, Sylvain Coulombe, Richard L Leask, Elizabeth AV Jones

**Affiliations:** 1 Chemical Engineering Department, McGill University, 3610 rue University, Montréal, H3A 2B2, Canada; 2 Montreal Heart Institute, Montréal, H1T 1C8, Canada; 3 Lady Davis Institute, Jewish General Hospital, McGill University, 3755 Cote Ste- Catherine Rd, Montreal, QC, H3T 1E2, Canada

**Keywords:** Transfection, Non-viral gene therapy, Naked plasmid DNA, Gene expression

## Abstract

**Background:**

Transferring genes and drugs into cells is central to how we now study, identify and treat diseases. Several non-viral gene therapy methods that rely on the mechanical disruption of the plasma membrane have been proposed, but the success of these methods has been limited due to a lack of understanding of the mechanical parameters that lead to cell membrane permeability.

**Methods:**

We use a simple jet of inert gas to induce local transfection of plasmid DNA both *in vitro* (HeLa cells) and *in vivo* (chicken chorioallantoic membrane). Five different capillary tube inner diameters and three different gases were used to treat the cells to understand the dependency of transfection efficiency on the dynamic parameters.

**Results:**

The simple setup has the advantage of allowing us to calculate the forces acting on cells during transfection. We found permeabilization efficiency was related to the dynamic pressure of the jet. The range of dynamic pressures that led to transfection in HeLa cells was small (200 ± 20 Pa) above which cell stripping occurred. We determined that the temporary pores allow the passage of dextran up to 40 kDa and reclose in less than 5 seconds after treatment. The optimized parameters were also successfully tested *in vivo* using the chorioallantoic membrane of the chick embryo.

**Conclusions:**

The results show that the number of cells transfected with the plasmid scales with the dynamic pressure of the jet. Our results show that mechanical methods have a very small window in which cells are permeabilized without injury (200 to 290 Pa). This simple apparatus helps define the forces needed for physical cell transfection methods.

## Background

Cell transfection has become a vital tool in the way diseases are studied and treated. Methods based on endocytosis [1], electroporation [2] and viral vectors [3] are well established and allow DNA, RNA and proteins to penetrate the cell membrane that would naturally be impermeable. Other methods are believed to rely on physical disruption of the plasma membrane by mechanical forces to facilitate macromolecule transport. Particle bombardment (such as the gene gun) shoots gene-coupled particles directly at the membrane [4]. Sonoporation is believed to use the forces created by acoustic waves to disrupt the lipid bilayer [5,6]. The scrape-loading method uses direct mechanical stress to crudely permeabilize cells [7]. More recently, fluid shear stress has been shown in microfluidic devices to permeabilize cultured cells [8]. Hydrodynamic gene delivery is thought to push DNA through the plasma membrane *in vivo* by injecting large amounts of solution containing DNA into a treatment area [9]. The precise mechanics of these mechanical methods are ill-defined but key to their success as the margin between transfection and cell damage may be small.

Though mechanical methods of transfection show considerable promise, the efficiency of transfection is low compared to viral transfection methods. Concerns remain, however, about the oncogenic potential or pro-inflammatory effects of viral transfection methods [10]. For these reasons, there is significant interest in improving the efficiencies of non-viral gene transfection techniques. Though in some cases, high transfection efficiency has been achieved using lipofection-based techniques, efficiencies are very dependent on the cell type and have been as low as 2% for some non-proliferating cell lines [11]. Mechanical transfection methods have also met with variable success. Using 1 MHz ultrasound transfection (with 20s exposure), which is equivalent to 0.1 – 0.5 MPa, Greenleaf *et al.* were able to achieve a 50% transfection efficiency in a chondrocyte cell line [12]. Koch *et al.* used a clinical spectral Doppler ultrasound system with 2 MHz frequencies and 90s exposure to obtain 32.7% GFP transfection efficiency *in vitro*, in comparison to 7.4% in their control [13]. Though the efficiencies achieved using mechanical methods can surpass the efficiencies obtained by lipofection, they do not come close to the efficiencies obtained using retroviral techniques that can be as high as 80% *in vitro*[14]. Only through a better understanding of the forces required to permeabilize cells by mechanical means can we hope to improve these results.

The efficiency of transfection techniques depends on many factors. Though the mechanical and energetic parameters are most often studied, other factors such as temperature [15,16], cell density, and cell lines [17,18] are also important. Many transfection techniques, such as laser-irradiation, are more efficient at higher temperatures [19]. Though temperature can be an important parameter for transfection efficiency, it is not feasible to alter temperature *in vivo*. Similarly, cell density has a significant effect on the efficiency of many transfection method, but is dictated by the tissue itself and cannot be controlled independently. Therefore, understanding the role of mechanical parameters on transfection efficiency offer the greatest potential for improving *in vivo* transfection.

Here we use mechanical forces produced by an inert gas jet to disrupt the lipid bilayer and facilitate cell transfection in adherent cell culture (*in vitro*) and into the chorioallantoic membrane (CAM) of the chicken embryo (*in vivo*) in a spatially specific manner. This simple set up allowed us to vary the jet parameters required to create pores in cell membranes and thereby calculating the important physical parameters leading to cell permeabilization. We used different inert gases, flow rates and glass capillary tube diameters to vary the magnitude of the physical force applied on a biological tissue. In addition, we used this system to estimate the pore size and the time period for which these pores remained open.

## Results

### Transfection efficiency dependence on mechanical parameters

**Figure 1:**
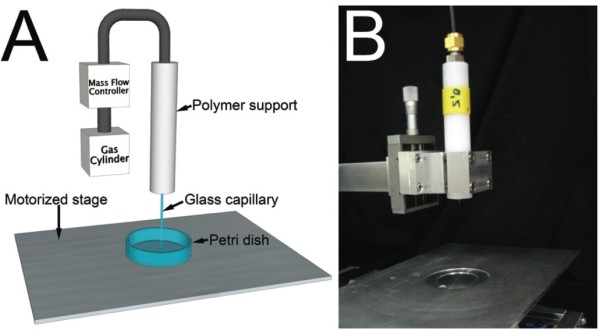
**Device used for transfection.** (**A**) Schematic of the device used for cell transfection and its components. (**B**) Picture of the device used for cell treatment positioned over the motorized stage.

**Figure 2:**
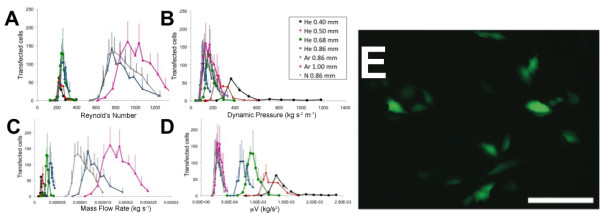
**Typical cell transfection curve as a function of the dynamic pressure.** (**A**), the Reynolds Number (**B**), the mass flow rate (**C**) and the viscosity of the gas multiplied by the velocity (**D**). All error bars represent the standard error obtained by counting 3 samples. (**E**) Typical picture of cells transfected with hrGFP-II 1 plasmid found along the treated pattern. The picture presented here was obtained using a capillary tube diameter of 1.00 mm and 0.675 L/min of argon. Scale bar 100 μm.

A simple device consisting of a standard glass capillary fixed to a plastic support (Figure 1) was used to treat an adherent monolayer of cells (HeLa) at various flow rates. To better understand the dependency of temporary cell permeabilization on the fluid parameters, five different capillary tube inner diameters and three different inert gases were used to treat the cells. We successfully transfected the cells using a 4.9 kbp hrGFPII-1 plasmid, leading to the expression of a green fluorescent protein. We counted the number of transfected cells (Figure 2A, B, C and D) under various flow conditions and using different gases. The results are expressed in terms of the number of transfected cells 24 hours post-treatment as determined by manual counting using fluorescence microscopy (Figure 2E).

To identify the critical fluid parameters that lead to temporary cell membrane permeabilization, the number of transfected cells was plotted as a function of the Reynolds number (ρVD/μ, where ρ is the gas density at 20°C, V is the jet average velocity, D is the inner diameter of the capillary and μ is the viscosity of the gas at 20°C), the dynamic pressure of the gas (ρV^2^/2), the mass flow rate (ρVπD^2^/4), as well as the viscosity multiplied by the velocity (μV). The typical response observed, for the number of transfected cells, passes through a maximum before decreasing at higher gas flow rates. When the data was plotted with respect to the dimensionless Reynolds number (Re), data from a given gas grouped together, independent of the capillary diameters (Figure 2A). Changing the gas, changed the peak efficiency Re. When expressed in terms of the dynamic pressure of the gas coming out of the capillary; the transfection curves for all gases appeared to group better, however there was a noticeable effect of the capillary diameter (Figure 2B). Other combinations of the fluid parameters did not produce better grouping than the dynamic pressure (examples Figure 2C and 2D). We therefore found the transfection efficiency to be best described by the dynamic pressure and that the range of dynamic pressure that leads to transfection was small for all configurations tested (200 ± 20 Pa).

### Cell stripping and cell death

**Figure 3:**
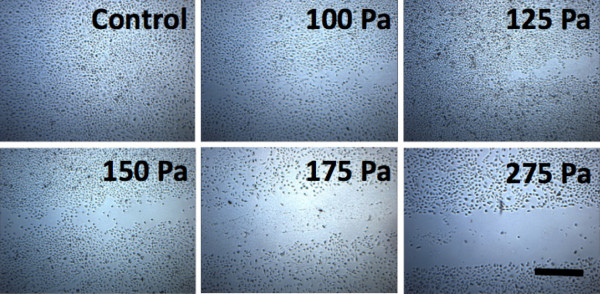
**Cell detachment observed as a function of gas flow.** Bright field picture of the upper right corner of the treated pattern for helium gas flow rates of 0.60, 0.75, 0.85, 0.93, 1.00 and 1.25 L/min and an inner capillary tube diameter of 0.68 mm using helium gas. Scale bar 500 μm.

**Figure 4:**
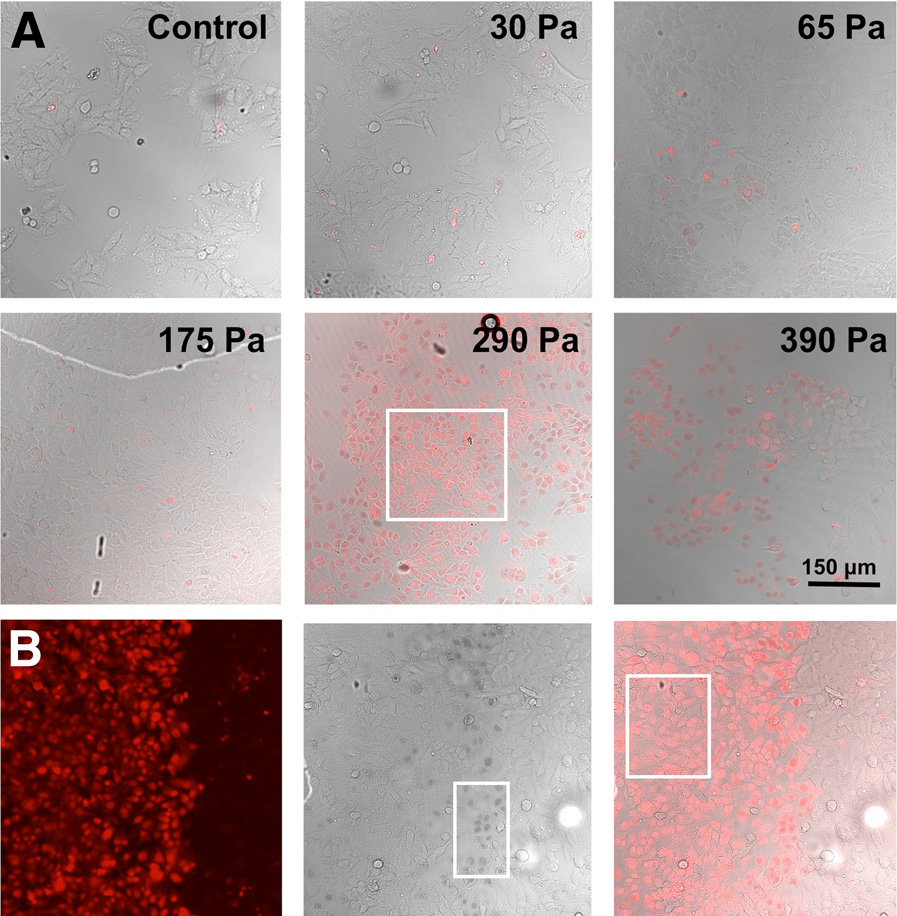
**Analysis of cell permeabilization and cell death as a function of gas flow.(A)** The cells were imaged on a fluorescent microscope after treatment with a helium gas jet exerting 0, 30, 65, 175, 290 and 390 Pa. Scale 150 μm. For this set of experiments, 500 μL of 120 μg / mL 10 kDa Texas Red dextran was used and a capillary diameter of 0.68 mm. The white box shows permeabilized live cells. Most of the cells in the 390 Pa image are dead. **(B)** From left to right, confocal image showing the fluorescence from the 10 kDa dextran molecules inside the HeLa the cells as a result of the helium gas jet treatment at 350 Pa (0.68 mm capillary diameter). Brightfield image of the same region, showing Trypan blue in the nucleus of a majority of cells which have died due to the treatment. Trypan blue is located in the nucleus of the cells, which appear darker as outlined by the white box. The composite image with the fluorescence and dead cell stain, indicates that a good number of living cells were permeabilized due to the treatment (box). Scale bars (A,B) 150 μm.

We observed that cell detachment, or stripping, occurred at the higher dynamic pressures (Figure 3). At the lower dynamic pressures tested, cell-sparse regions at the centre of the gas jet were observed. At high dynamic pressures (>275 Pa), the entire region where the gas jet was applied was devoid of cells. Since the hrGFPII-1 plasmid can only be expressed in live cells, we used fluorescent dextrans (10 kDa, 120 mg/mL) to identify permeabilized cells and Trypan blue to identify cell death. Experiments were done with a 0.68 mm capillary tube diameter and a range of gas flow rates generating up to 450 Pa at the surface of the cells (Figure 4). In the control samples, very little fluorescence was detected, with dim fluorescence gradually appearing as the dynamic pressure head was increased up to 175 Pa. At 275 Pa (Figure 4A), all cells on the path of the gas jet were detached and peak permeabilization was found at approximately the same dynamic pressure head (290 Pa). No trypan-blue positive dark nuclei were observed below 290 Pa. The permeabilized cells were densely located at the outskirts of the gas jet. Dynamic pressure above 290 Pa killed a significant portion of the cells (Figure 4B). At a dynamic pressure of 390 Pa, the majority of permeabilized cells were dead. Therefore a dynamic pressure of 290 Pa led to permeabilization without cell death. Above this pressure, a significant number of permeabilized cells stained positive for Trypan Blue indicating cells were dying.

### Permeabilization time course

**Figure 5:**
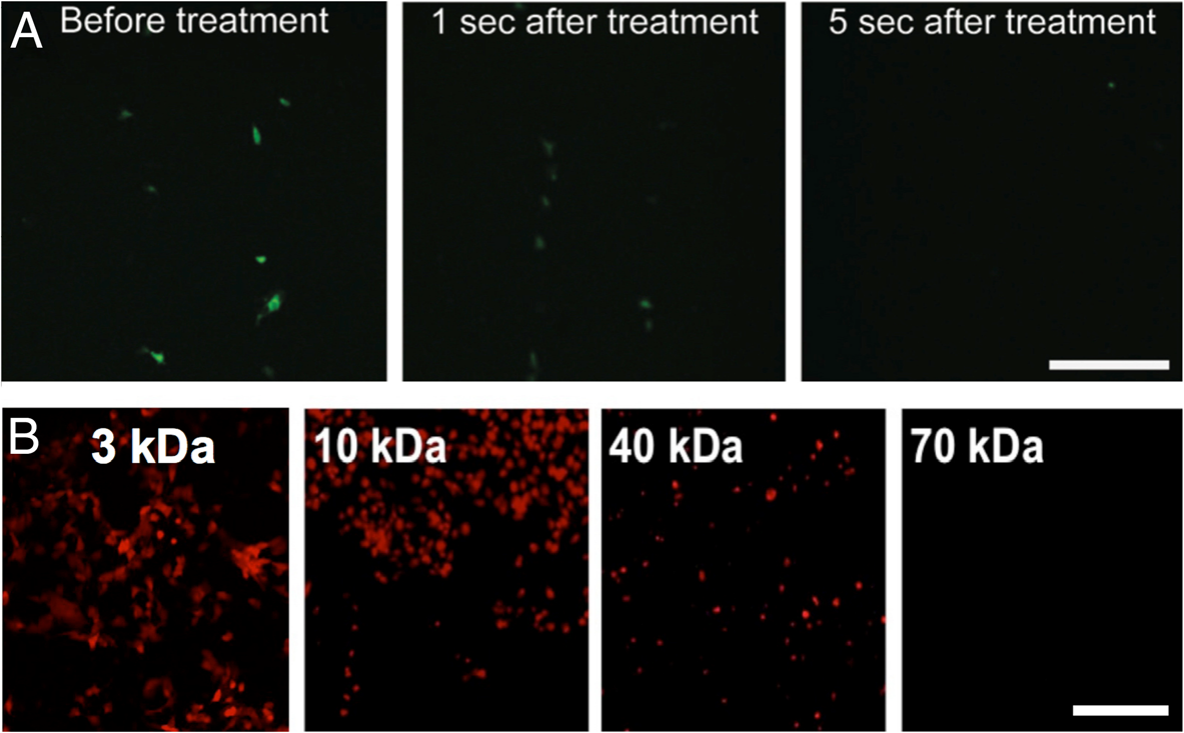
**Pore size and duration during permeabilization.** (**A**) Time course study. The 40 kDa dextran solution was added before treatment, 1 second after treatment or 5 seconds after treatment. Dextran molecules are membrane impermeant. Fluorescent cells indicate that the membrane of the cell was permeabilized at the time the solution was added. (**B**) Size exclusion study. Dextran molecules of 3 kDa, 10 kDa (1.9 nm hydrodynamic radius), 40 kDa (4.8 nm hydrodynamic radius) and 70 kDa (6.5 nm hydrodynamic radius) were added to the media before treatment. Cells were treated with helium gas, using a capillary diameter of 0.68 mm and a flow rate of 1.3 L/min for all dextrins (297 Pa). No fluorescence could be detected using 70 kDa dextran molecules. Fluorescent cells indicate that the membrane of the cell was permeabilized. Scale bar (A,B) 200 μm.

In order to investigate the dynamics of pore formation, fluorescent dextran molecules were used to perform a time course and a size exclusion studies. Dextran was added to the solution at various times after the gas treatment. Dextran added before treatment or one second after could be taken up (Figure 5A). There was no fluorescence detected when the dextran molecules were added 5 seconds after the treatment. We then used dextrans of different molecular weight to estimate pore size. Figure 5B shows that dextran molecules up to a size of 40 kDa could be introduced in the treated cells. The introduction of dextran molecules of 70 kDa was also attempted but no fluorescent cells were detected.

### Demonstration of *in-vivo* transfection

**Figure 6:**
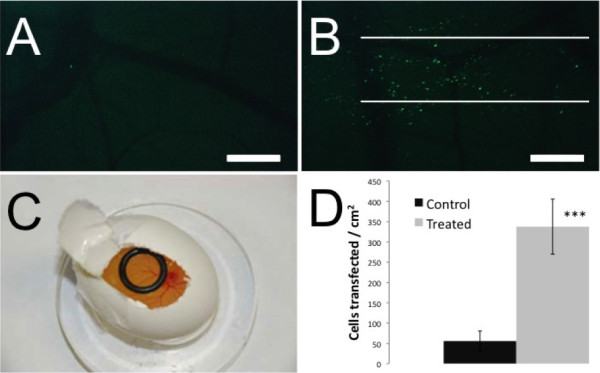
**Transfection of a chicken embryo using optimized parameters.** (**A**) Epifluorescence picture of a control chicken embryo 24 hours following treatment with the hrGFP-II 1 plasmid. (**B**) Epifluorescence picture of a chicken embryo 24 hours following gas treatment with the hrGFP-II 1 plasmid and helium gas at a flow rate of 1.5 L/min. The white lines show the treatment path of the 1 mm diameter capillary tube. (**C**) Setup of the chicken embryo for the gas flow treatment. The o-ring used to contain the treatment solution is positioned on top of the CAM. (**D**) The number of transfected cells was counted blindly and the results show a six-fold increase in transfection efficiency with a helium gas treatment at 2.5 L / min (n = 3 for both control and experimental, P < 0.0005). Scale bars (A,B) 1 mm.

Finally, to demonstrate the potential of a simple inert gas flow for *in vivo* transfection, we used the chicken chorioallantoic membrane (CAM) as a tissue model (Figure 6). The plasmid solution was added by placing an o-ring over the treated region before exposure to the gas jet (Figure 6C). An average of 340 GFP expressing cells per cm^2^ were observed following a gas treatment with helium at 1.5 and 2.5 L/min using a capillary of 1.00 mm in diameter (Figure 6B). In control experiments (addition of plasmid solution without gas flow), only 55 cells per cm^2^ expressed GFP. Therefore, a 6-fold increase (n = 3 for both control and experimental, P < 0.0005) was found in the number of cells transfected between the treated embryos and control embryos (Figure 6D). The inclusion and expression of the plasmid was confirmed by counter-staining with an anti-GFP monoclonal antibody (data not shown).

## Discussion

In this work, we analyse mechanical parameters that are required for temporary cell permeabilization using a gas jet. We found that the efficiency of transfection increased with increasing gas flow rate. At the same time, increasing the gas flow rate increased the frequency of cell detachment and cell death. Therefore, our data demonstrate that there is a fine balance between temporary cell permeabilization, cell death and stripping of the cells that is dependent on the flow parameters of the jet. We then show that the optimized parameters can effectively transfect cells *in vivo*, using the chorioallantoic membrane of chick embryos.

We used five different capillary inner diameters and three different inert gases in order to investigate the dependency of the number of transfected cells on the jet area, flow rate and gas material properties (density and viscosity). The number of transfected HeLa cells with the hrGFPII-1 plasmid was compared with measures of the gas jet dynamics. The Reynold’s number is a measure of the inertial forces of the gas versus the viscous forces. It is predictive of the internal flow physics of the jet. As can be observed in Figure 2A, plotting the number of transfected cells versus the Re regroups the curves obtained for each gas but there was a divergence present when the curves are compared for different gases. The same pattern was obtained using the mass flow rate (Figure 2C) and the viscosity multiplied by velocity (Figure 2D). We hypothesized that the energy of the jet impinging the culture media covering the cells was responsible for the temporary permeabilization and used a simplified measure of jet dynamics, the dynamic pressure (ρV^2^/2). The results showed a grouping of peak transfection independent of gas used at a dynamic pressure of 200 ± 20 Pa for HeLa cells (Figure 2B).

The results provide good evidence that there is a strong relationship between the dynamic pressure and the number of cells transfected. The smallest inner diameter capillary required a higher dynamic pressure to achieve peak efficiency, however, suggesting that there are other dependent variables not accounted for by the dynamic pressure alone. It is also evident that a greater number of transfected cells are obtained with the larger diameters capillary. This is most probably due to the larger number of cells treated with the larger jet.

Our estimated level of stress (dynamic pressure 200 ± 20 Pa) is similar to estimated levels of fluid shear stress shown to permeabilize cells in flow channels. Hallow *et al.* demonstrated temporary permeabilization of suspended cells in small channels under short bursts of flow [8]. Steady flow simulation using computational fluid dynamics (CFD) estimated the optimal shear to be around 200 Pa (with no cells). Unfortunately, the efficiency of transfer was low and cell viability significantly decreased with channel diameter. The same authors used a cone-plate rheometer to permeabilize adherent cells with a short (300 μs) impulse of 140 dynes/cm^2^. The efficiency was low and the authors did not distinguish between temporary and permanent (dead) pores. Using micro-particle image velocimetry (μPIV) and high speed imaging, Sankin *et al.* were able to capture pores on the surface of adherent cultured cells in the path of the micro-jet created by the collapse of laser-generated tandem micro-bubbles [20]. Using μPIV-extrapolated jet velocities the authors estimated maximal shear stresses around 1000 Pa caused pore formation in their cell line. The authors concluded that “flow shear stress and resultant bending (and stretching) of the cell body may contribute to the observed pore formation” [20].

Ohl *et al.* showed shock waves directed at cultured cells can produce cavitation bubbles [21]. The collapse of these bubbles is intense and can detach nearby cells. They found that cells on the borders of the crater made in the monolayer were permanently permeabilized (dead). Just outside the impact zone, temporary permeabilization was observed. Stripping of the cells occurred at stresses estimated above 160 Pa when applied for short durations (~1 μs). No attempt was made to relate the magnitude of shear stress to temporary pore formation.

We found cell stripping to occur at dynamic pressures greater than 275 Pa (Figure 3). Others have shown that the fluid shear stress needed to detach WT NR6 fibroblast cells is between 200 and 267 Pa [22]. We also evaluated the extent of cell death in permeabilized cells using 10 kDa dextrans and Trypan blue. From Figure 5B, one can see that all permeabilized cells are dead at 390 Pa. Like Ohl *et al.*[21] the cells closest to the borders of detachment were dead, while cells further away were temporarily permeabilized.

The efficiency of the hydrodynamic gene delivery method has been related to the fluid injection’s volume, velocity and pressure [23-26] and it is interesting to note that the optimal value for the dynamic pressure obtained in our experiments (200 ± 20 Pa) falls into the range of values we have calculated from the published data for hydrodynamic gene delivery (3 – 1200 Pa) [23,26-28]. The wide range of values calculated from literature data might be explained by the fact that different conditions were used. In most experiments, the liquid was injected inside different veins [26-28]; therefore the hydrodynamic force was not directly applied to the cells. In another experiment, the liquid was injected directly inside a muscle [23] hence the distance between the injection point and the treated cells was minimal. The possibility that the DNA could enter the cell by impact similar to the gene gun has been ruled out since the impact stress used in the gene gun is considerably higher (~344 kPa) [29].

Despite the simple and controlled setup, the overall dynamics of this problem are still very complex, involving the interaction of the gas with the liquid and potentially with the surface of the cells. It is, however, clear that the forces created by the jet are involved in the temporary permeabilization of the cells. The dynamic pressure provides a rough estimate of the resulting stress on the plasma membrane created by the displacement of the media and the direct pressure of the jet applied to the cells. This stress is hypothesized to cause the temporary disruption of the plasma membrane (< 5 s, Figure 5A) allowing macromolecules such as the hrGFPII-1 plasmid and dextran (up to 40 kDa, Figure 5B) to diffuse into the cell.

## Conclusions

The results show that the number of HeLa cells transfected with the hrGFPII-1 plasmid scales with the dynamic pressure of the jet. We find that the range of dynamic pressure that effectively permeabilizes cells without injuring them is small (200–290 Pa). We further show that these optimized conditions can successfully transfect cell *in vivo* using chicken embryos. Together, these results help define the forces that must be generated for mechanical cell transfection methods.

## Methods

### Experimental setup

A mass flow controlled jet of inert gas was used to temporarily permeabilize cells. The device consisted of a motorized stage, glass capillary tubes (0.40, 0.50, 0.68, 0.86 and 1.00 mm in inner diameter), a polymer support, a gas mass flow controller, regulator and cylinder (Figure 1). Three different gases (Ar, N_2_ and He) were used in the experiments with different densities and viscosities. The density of Argon (Ar) is 1.662 kg/m^3^ and the viscosity is 2.10 × 10^-5^ Pa.s at STP conditions. Nitrogen (N_2_) has a density 1.131 kg/m^3^ and viscosity of 1.67 × 10^-5^ Pa.s. Helium at STP. Helium (He) has a density of 0.1667 kg/m^3^ and viscosity of 1.86 × 10^-5^ Pa.s at STP conditions. The flow rate of the gas (from 0 to 2.5 L/min), speed of the motorized stage (5 mm/s) and dish diameter (12 or 35 mm) were controlled through a NI-LabVIEW^TM^ program. The dynamic pressure of the jet was crudely estimated from the average velocity of the gas (from the flow rate and capillary tube area) and density of the gas at room temperature. Better estimates of the force applied to the cells would require detailed computational and experimental studies involving both the gas and liquid covering the cells.

### Cell culture

HeLa cells (ATCC #CCL-2) and culture products were purchased from ATCC. The cells were cultivated in minimum essential medium eagle (MEME) supplemented with 10% foetal bovine serum, 100 μg/mL of streptomycin and 100 μg/mL of penicillin. For the transfection study, cells were expanded in 100 mm diameter Petri dishes (Sarstedt #83.1802.003) and incubated at 37°C with 5% CO_2_. The day prior to cell treatment, the cells were plated at a density of 10^5^ cells per well in 12-well plates (Corning #3512). Each well was coated for 4 hours in the incubator using 240 μL of a 25 μg/μL collagen (Sigma #C5533) solution. For the dextran uptake and cell death study, cells were expanded in T175 culture flasks and incubated at 37°C with 5% CO_2_. Two days prior to cell treatment, the cells were plated at a density of 2 × 10^5^ cells per well in 6-well plates (Corning #3335). Each well was coated for 1 hour in the incubator using 500 μL of a 0.1% collagen (Sigma #C5533) solution prior to seeding.

### Adherent cells transfection

The human recombinant green fluorescent protein plasmid (hrGFP-II-1) was purchased from Stratagene (#240143) and produced in E. coli DH-1 (ATCC #33849) competent cells. The plasmid was purified using Qiagen Plasmid Giga kits (#12191).

Before performing the gas treatment, the cells were rinsed with 1 mL of 0.5xPBS. Following the rinse, 500 μL of 0.5 × PBS containing 25 μg of hrGFP II-1 plasmid (Stratagene) was added to each well. The gas treatment was performed 1 minute after the addition of the plasmid solution. The distance between the end of the capillary tube and the bottom of the well was set to 3 mm [30]. The displacement speed of the motorized stage was set to 5 mm/s. The treated pattern consisted of a rectangle of 11.3 mm × 5.7 mm (~ 10^4^ cells). Immediately after treatment, the plasmid solution was removed and 1 mL of fresh media was added to the well. Cells were incubated for 24 hours before counting and imaging under fluorescence microscopy (Leica Microsystems 090–135.002 equipped with DC 300 V.2 camera). Three measurements were made per setting and the standard error of the measurements is reported.

### Trypan blue staining

Before performing the gas treatment, the cells were rinsed three times with 1 mL of 1 × PBS. Following the rinse, 500 μL of 1 × PBS containing 60 μg of 10 kDa Texas Red dextran (Molecular Probes #D1828) was added to 3 of the 6 wells. Gas treatment was performed as described for transfection experiments. Immediately after treatment, the dextran solution was removed, the cells were then rinsed three times with 1 × PBS and 1 mL of fresh media was added to the well. Cells were incubated for 30 minutes before counter staining with 0.4% Trypan blue and fixing with a 1% paraformaldehyde solution.

### Dextran molecules time course

Before the experiment, the growth media was removed and 500 μL of 0.5 × PBS was added to the cells. Gas treatment was performed as described for transfection. 40-kDa fluorescein dextran (Molecular Probes #D1844) was added either before treatment, 1 second after treatment or 5 seconds after treatment to a final concentration of 80 μg/μL. The treated cells were rinsed three times with media and immediately imaged using fluorescent microscopy (Leica Microsystems 090–135.002 equipped with DC 300 V.2 camera).

### Dextran molecules size exclusion

Before the experiment, the growth media was removed and 500 μL of 1 × PBS containing 80 μg/μL of 3, 10, 40, or 70 kDa Texas Red dextran (Invitrogen #D3329, #D1828, #D1829 and #D1830) was added. Gas treatment was performed as described for the transfection experiments. Immediately after gas treatment, the solution was removed and 1 mL of fresh media was added to the treated cells. The treated cells were rinsed three times with media and immediately imaged using fluorescent microscopy (Leica Microsystems 090–135.002 equipped with DC 300 V.2 camera). The size of the pores was estimated based on published values for the hydrodynamic radius of the dextran molecules [31].

### Chorioallantoic membrane (CAM) transfection

The chorioallantoic membrane (CAM) of day 7 chick-fertilized embryos was exposed by making a window in the egg shell, and an o-ring was then placed directly on the CAM (Figure 6C). Three hundred (300) μL of plasmid (0.05 mg/mL) in 1 × PBS was pipetted into the o-ring. The CAM was treated with the gas flow by positioning the tip of the capillary approximately 3 mm from it. The displacement speed of the motorized stage was set to 5 mm/s. The treated pattern consisted of one set of parallel lines of 15 mm in length and separated by 0.5 mm. The o-ring was then removed and the eggs resealed and re-incubated for 24 hours. The presence of fluorescent cells was scored under a fluorescent dissecting scope, blind to treatment. Control experiments consisting of a gas flow jet without the addition of the plasmid solution, the addition of the plasmid solution without gas treatment and untreated eggs were also performed. Statistical significance was assessed using two-tailed student’s *t*-test.

### Immunohistochemistry

For whole-mount staining, CAMs were fixed in 4% paraformaldehyde overnight at 4°C and stored in methanol at −20°C. Embryos were progressively rehydrated and blocked twice for 1 hour in TNB, which is the Tris-NaCl-Blocking Buffer (Roche #11096176001). Embryos were incubated overnight at 4°C with primary anti-GFP antibodies (1:100) (Stratagene #240241) in TNB. Embryos were washed, re-blocked and incubated overnight with AlexaFluor-555 conjugated secondary antibodies (1:400) (Invitrogen #A31570) at 4°C. Samples were washed, mounted and imaged on a Zeiss EXCITER confocal microscope with a 40 x objective lens. The red and green channels were imaged sequentially to prevent cross-excitation.

## Competing interests

The authors declare that they have no competing interests.

## Authors' contributions

GCP performed the experiments on permeabilization to dextrans, cell stripping, cell death and *in vivo* transfection as well as contributing the writing of the manuscript. ML and DG designed the gas jet apparatus and did the transfection experiments with the GFP plasmid. SC contributed to the design of the gas flow apparatus. RLL supervised the research, contributed to the design of the gas flow apparatus, as well as performing analysis of results and contributed to the writing of the manuscript. EAVJ supervised the research, contributed to the analysis of cell permeabilization, *in vivo* transfection, as well as performing analysis of results and contributed to the writing of the manuscript. All authors read and approved the final manuscript.
